# VCGDB: a dynamic genome database of the Chinese population

**DOI:** 10.1186/1471-2164-15-265

**Published:** 2014-04-05

**Authors:** Yunchao Ling, Zhong Jin, Mingming Su, Jun Zhong, Yongbing Zhao, Jun Yu, Jiayan Wu, Jingfa Xiao

**Affiliations:** 1 CAS Key Laboratory of Genome Sciences and Information, Beijing Institute of Genomics, Chinese Academy of Sciences, Beijing 100101, China; 2 University of Chinese Academy of Sciences, Beijing 100049, China; 3 Computer Network Information Center, Chinese Academy of Sciences, Beijing 100190, China

**Keywords:** Chinese population, Dynamic genome, Database, 1000 Genomes Project, Big data

## Abstract

**Background:**

The data released by the 1000 Genomes Project contain an increasing number of genome sequences from different nations and populations with a large number of genetic variations. As a result, the focus of human genome studies is changing from single and static to complex and dynamic. The currently available human reference genome (GRCh37) is based on sequencing data from 13 anonymous Caucasian volunteers, which might limit the scope of genomics, transcriptomics, epigenetics, and genome wide association studies.

**Description:**

We used the massive amount of sequencing data published by the 1000 Genomes Project Consortium to construct the Virtual Chinese Genome Database (VCGDB), a dynamic genome database of the Chinese population based on the whole genome sequencing data of 194 individuals. VCGDB provides dynamic genomic information, which contains 35 million single nucleotide variations (SNVs), 0.5 million insertions/deletions (indels), and 29 million rare variations, together with genomic annotation information. VCGDB also provides a highly interactive user-friendly virtual Chinese genome browser (VCGBrowser) with functions like seamless zooming and real-time searching. In addition, we have established three population-specific consensus Chinese reference genomes that are compatible with mainstream alignment software.

**Conclusions:**

VCGDB offers a feasible strategy for processing big data to keep pace with the biological data explosion by providing a robust resource for genomics studies; in particular, studies aimed at finding regions of the genome associated with diseases.

## Background

The 1000 Genomes Project Consortium has used the dramatic increase in sequencing power that has become available to sequence the genomes of 1092 individuals from 14 populations in different parts of the world [1,2]. Other approaches, like genome-wide association studies (GWAS), combine several hundred thousand variants from different individuals known to have a particular disease and related clinical traits, thereby associating genome-wide genotyping with the phenotypic disease for gene discovery [3,4]. The amount of healthcare-related data that are being digitally collected and stored, especially disease-related sequence variations that are used widely in personal medicine studies, are vast and expanding rapidly [5,6]. As a result, data management and analysis tools to convert this vast resource into information and knowledge are also advancing [7,8]. Third-generation high-throughput sequencing technology with its extraordinarily higher throughput and lower cost is now available [9,10]. Meanwhile, traditional sequencing platforms are still producing about five petabytes of sequencing data annually. These two technologies are driving the exponential growth of the genomics data "ocean" [11-13], which raises urgent problems on how to handle such huge amounts of data, including their storage, transfer, integration, and mining [14-18]. Sequencing data from different sample preparation protocols and various sequencing platforms with variable read lengths and sequencing coverage are often handled with different analysis tools and parameters. Thus, the standardization of sequencing studies and their interpretation are challenges that researchers are beginning to pay more attention to [19].

The current Genome Reference Consortium human genome (build 37), GRCh37, is derived from 13 anonymous Caucasian volunteers from Buffalo, New York [20]. This sequence is used as a standard template and a guiding principle for the discovery of low-frequency variants in different individuals from different populations, the development of computational software, and for building clinical genomic resources [21,22]. Although the reference genome has been revised several times, it still offers limited information for the study of population- and individual-specific variants. The human reference genome sequence is unable to meet the precise investigative requirements of genomics, transcriptomics, epigenetics, and genome-wide association studies (GWAS) [23]. Several efforts have been made to generate specific complete human genome sequences of different populations. Levy *et al.* reported the whole genome sequence of a Caucasian individual of Western European ancestry (CEU) sequenced using Sanger methodology [24]. Bentley *et al.* used the short reads sequenced on a next-generation sequencing platform to determine the genome sequence of a Yoruba individual from Ibadan, Nigeria (YRI) [25]. Wang *et al.* sequenced the genome of a Han Chinese individual using combined strategies for alignment and assembly [26]. Other population-specific genome sequences, including the genomes of a Korean and an Irish individual, have been released [27,28]. Each of these genome sequences represent individuals from a particular population and are annotated with information about single nucleotide polymorphisms (SNPs), insertions/deletions (indels), and large structure variations based on the human reference genome sequence. These data are for one individual’s genome only and do not represent or characterize population-specific differences [29].

The huge amount of human genome sequence data [30-32] has made it possible to detect high- and low-frequency variants across the whole human genome. As a result, the focus of human genome studies are changing from single and static to complex and dynamic [33]. The 1000 Genomes Project Consortium has generated and published a massive amount of human genome sequencing data. Using some of this data, we have constructed the Virtual Chinese Genome Database (VCGDB), a dynamic genome database of the Chinese population based on whole-genome sequencing of 194 individuals. VCGDB is "virtual" because the reference genome provided in the database is the statistical result of terabases of sequencing data from hundreds of Chinese individuals that describe the genetic variation features specific to the Chinese population. VCGDB is "dynamic" because dynamic variations of individual characters and genomic annotation information, such as reference genes, genomic duplications, and GWAS clinical traits are integrated in the database. VCGDB offers a strategy for processing large amounts of genomic data and is a robust database for genomics and disease-related studies in the Chinese population.

## Construction and content

**Figure 1:**
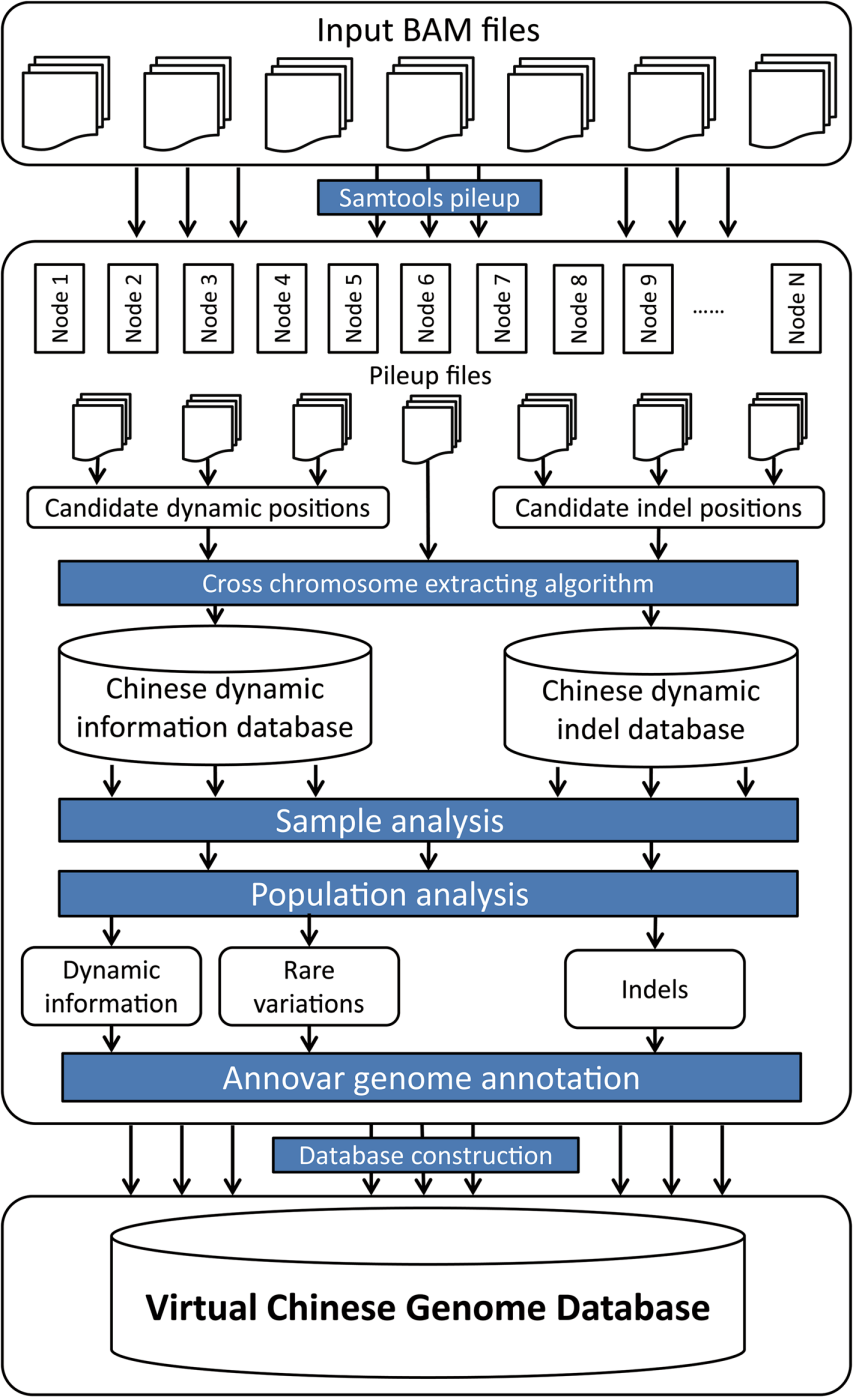
Data processing workflow used to construct the virtual Chinese genome database (VCGDB).

To handle terabase amounts of data, we strictly organized the data processing workflow and optimized the algorithms to run using limited calculating resources (Figure 1). The workflow was divided into six steps: data preprocessing, candidate dynamic information extraction, virtual genome dynamic information statistics, dynamic information validation, virtual genome annotation, and database construction.

### Data sources

We collected all the whole genome low-coverage (2–4x) alignment results of 194 individuals from two typical Chinese populations released by 1000 Genomes Project. The data included samples from 100 Southern Han Chinese individuals (CHS) and samples from 94 Han Chinese individuals in Beijing (CHB). All the data can be downloaded from mirror FTP sites of NCBI ( ftp://ftp-trace.ncbi.nih.gov/1000genomes/ftp/) or EBI ( ftp://ftp.1000genomes.ebi.ac.uk/vol1/ftp/). The sequencing data were generated in different laboratories using a variety of different platforms; therefore, all the data were standardized based on "Bavarva Theory" [19]. The raw sequencing reads have been mapped to the human reference genome using a consensus strategy based on different sequencing platforms, and the output is in binary BAM format [34]. The raw data and alignment data have been compressed to about 3.3 and 4.8 terabytes, respectively.

### Data preprocessing and candidate dynamic information extraction

In the data preprocessing step, we used SAMtools to multi-pileup all the samples to convert read-based alignment information into position-based data by splitting the reads into bases [34]. The process was optimized by piling up the samples in parallel. The resulting files from the 194 samples were about 70 gigabytes each and 12.6 terabytes in total.

The raw sequence data of the 194 whole genomes contained 3 billion nucleotide positions; therefore, a specific algorithm was required to extract meaningful information in a short time using limited storage space. We designed a two-step candidate dynamic information-extraction strategy to filter out irrelevant or redundant data and select the data for further data analysis and database construction. First, we built a candidate dynamic position list containing all the positions with variant probability within at least one sample. Assuming the samples to be independent from each other, we ran a parallel search and calculated the candidate dynamic positions (CDPs) from all the samples simultaneously. As a result, we obtained a non-redundant CDP hash dataset with a total of 55,549,120 CDPs from the 194 Chinese genome samples. Next, we developed a cross-chromosome data searching and extracting algorithm (Additional file 1: Figure S1) to filter the redundant data. The output of this process contained the mapped nucleotide information from each CDP together with the sample information. This candidate dynamic information-extraction strategy reduced the size of the data by 98%, which significantly decreased the programming difficulty and CPU time required for subsequent analysis.

### Virtual genome dynamic information statistics

The common SNP/SNV (single nucleotide polymorphism/single nucleotide variation) calling algorithms generally use parameters such as read quality to evaluate and filter variations caused by sequencing errors. This strategy is useful for sequencing data with a reasonable level of coverage from a single individual, because false positives of base positions will be quite low and differentiation between individuals does not need to be considered. However, for the massive amounts of low-coverage sequencing data from the large number of samples from different individuals in VCGDB, false positives could be higher and harder to detect, and difficult to distinguish from normal variations in each sample. Further, the samples were generated using different platforms under slightly different conditions, which may cause unequal weighting when the data are merged. To address these problems, we developed a data analysis strategy especially for large-scale data samples with low coverage, to preserve the independence and maintain equal weighting of each sample when handling the dynamic variation information.

First, we considered each base position on each sample as a unit; every selected unit had to have at least two mapped reads to ensure the accuracy of the sequencing. We used the comentropy of each unit to determine which nucleotide was dominant for that unit. Comentropy is a parameter that can be used to measure the uncertainty of the information in a thermodynamic system [35]. Here, a high comentropy value indicated that more information was included and the consensus of the data in the corresponding unit was less determinate. The comentropy equation was as follows:

$$H\left(x\right)=E\left[{\text{log}}_{2}^{p\left(x_{i}\right)}\right]=-\overset{n}{\underset{i=1}{\sum }}p\left(x_{i}\right){\text{log}}_{2}^{p\left(x_{i}\right)}$$

where, *x*_*i*_ is one of the detected nucleotide types (A/T/G/C); *n* is the number of detected nucleotide types; and *p(x*_*i*_*)* is the occurrence probability of each nucleotide type in a given unit. The units were filtered by their comentropy values against a threshold of binary unit ≤0.95, ternary unit ≤1.50, and quaternary unit ≤1.92.

Second, we calculated the nucleotide distribution of each CDP in three pre-defined populations, Han Chinese in Beijing (CHB), Southern Han Chinese (CHS), and an integrated dataset of CHB and CHS to represent the entire eastern region of China (CHN), and then used several parameters such as read depth, nucleotide distribution, major allele probability, and the population-level comentropy value to estimate each dynamic position in the corresponding population. Rare variations and indels are generally considered to have a high probability of being related to diseases and they are often the main factors that cause differences in genome size among different individuals or populations. We extracted rare variants that occurred in less than 5% of the samples and grouped them by population.

### Virtual genome annotation

Based on the annotations for the human reference genome sequence, we annotated the dynamic positions in the genome sequences of the three pre-defined Chinese populations to coding regions, intergenic regions, introns, and untranslated regions (UTRs). Traditional genome annotation databases and software such as the Gene Ontology and the KEGG pathway databases require a gene list as input and not position information, which makes them unsuitable for the dynamic positions and indels analysis that is required for the virtual genome data. Therefore, we used ANNOVAR, a position- and short region-based genome annotation software, to annotate the dynamic positions and indels in our data, especially the major allele and indel positions against the reference genome (hereafter referred to as MAIR) [36]. A number of databases, such as RefGene, genomicSuperDups, and gwasCatalog, were used to assign the annotations and an enrichment analysis was performed to reveal the potential biological significance of the dynamic genomics information [37].

### Database construction

To handle the huge amounts of data that were generated, we used the MySQL database management system to construct VCGDB. We configured the optimal relationships among the tables and in the data structure to obtain the best searching efficiency. The information obtained from the analysis described above was stored in VCGDB in three parts; dynamic position information, indel information, and genomic annotation information. The three parts were all highly structured, indexed, classified, and properly stored in MySQL tables. The chromosome name and the position index were used to build connections between the tables. The tables were split by chromosome name and every table was constrained to less than ten million records, which largely reduced the search region and the response time. Considering the data characteristics and search requirements, we constructed VCGDB in four main parts: "entity", "annotation", "dynamic", and "reference". The "entity" part stores basic information that describes the base positions; the "dynamic" part stores all the information that corresponds to dynamic positions, indels, and rare variations; the "annotation" part contains raw datasets of the annotation database associated with position-based annotation records; and the "reference" part is a structured database of linear references capable of high performance sequence initiation. We used the MAIR information to build population-specific consensus Chinese reference genome sequences named the virtual Chinese reference genomes (VCGs), for the three pre-defined populations and tested them by aligning raw sequencing data to the different genomes and comparing the mapped reads. The VCGs can be recognized by genome alignment software like BWA or Bowtie [38,39].

### Virtual genome visualization

Traditional genome browsers are not designed to visualize the dynamic genomics information in VCGDB. Therefore, we developed the Virtual Chinese Genome Browser (VCGBrowser), a highly flexible, region-based genome browser to display the virtual and dynamic genomes in VCGDB. VCGBrowser was built using the Java Swing Applet and the Genoviz software development kit (Genoviz SDK), an open source library of re-usable components for genomics data visualization [40]. VCGBrowser can be installed as a client-based application running in a personal computer, or it can be used online through the Java applet web interface. To ensure data transfer security, we have implemented a servlet so that users do not have to change their local security policy for a remote connection. The object-based methodology in VCGBrowser accelerates the transfer speed of visualized data (Additional file 1: Figure S2). Compatible testing was approved under mainstream operation systems like Windows XP, Windows 7, Ubuntu 12.04, and CentOS 6.0 and in browsers like Internet Explorer, Mozilla Firefox, and Google Chrome.

### Database content

**Figure 2:**
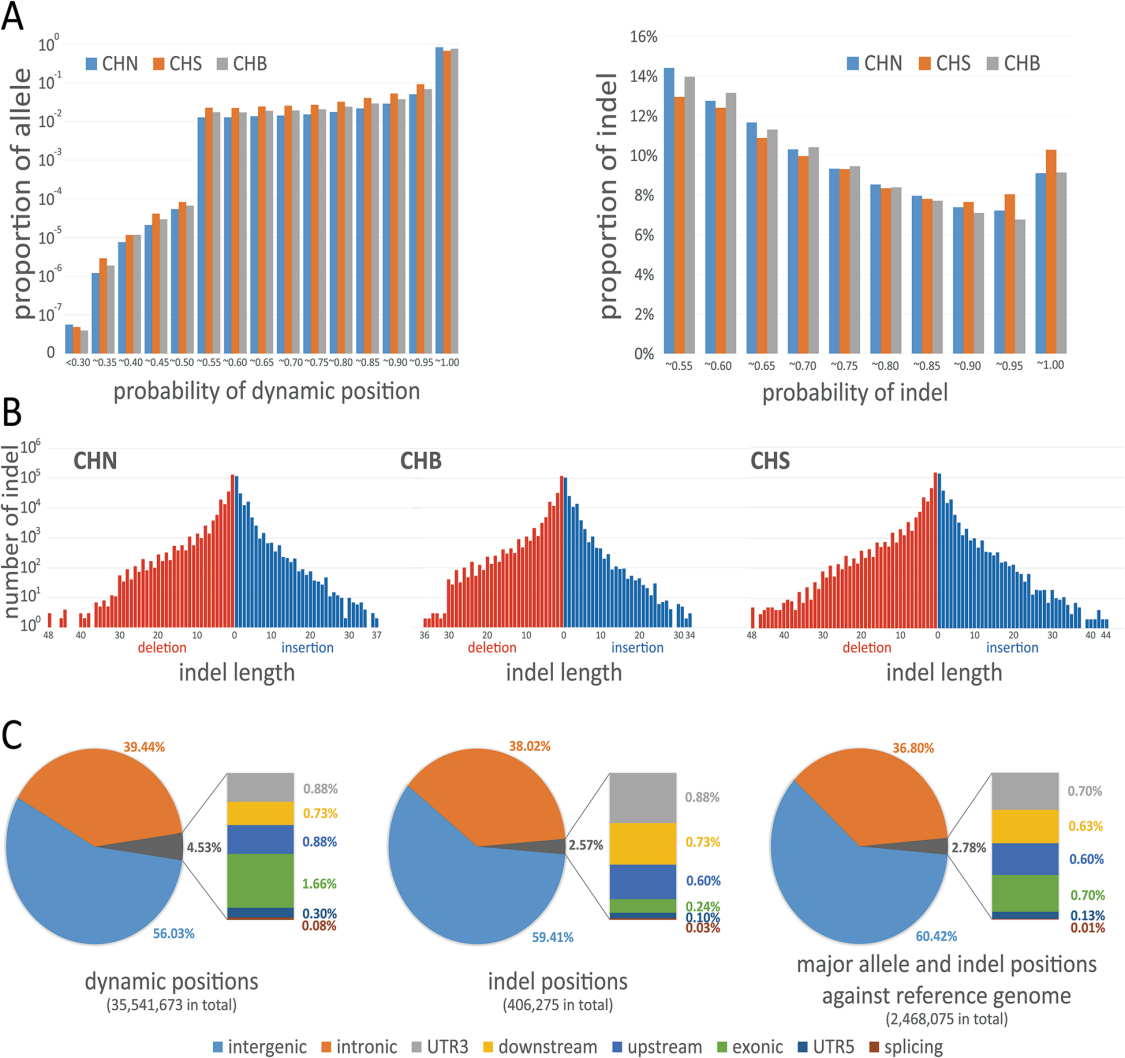
**Statistical analysis of the dynamic genomics information in the virtual Chinese genome database (VCGDB). A**. Dynamic positions and indel distribution in the CHN, CHB, and CHS populations. The X-axis shows the major base probability of the dynamic position/probability of indels in the genome sequences. The Y-axis shows the proportion of dynamic positions/indels with the specific probability region. **B**. Indel length distribution in the CHN, CHB, and CHS populations. The X-axis shows the length of insertions (blue) and deletions (red), and the Y-axis shows the number of indels. Only high-probability insertions and deletions (>50%) in VCGDB were counted. **C**. Distribution of dynamic positions, indels, and MAIR (major allele and indel positions against the GRCh37 reference genome) based on the annotation information.

After multi-level filtering and analysis, all the dynamic information for the three pre-defined populations, CHB, CHS, and CHN, was organized in 129 database tables. Several specific terms were defined to interpret the dynamic genomics information. A dynamic position was defined as a base position with nucleotide variation in the genomes of certain populations compared with the corresponding position in the GRCh37 reference genome. This situation can arise under four circumstances: one, major allele for a base position in a population that is different from the base in the corresponding position in the GRCh37 reference genome; two, indel position with a high probability (no less than 50%) in a population compared with the GRCh37 reference genome; three, variation for a base position in a population with at least one minor allele with low probability (but more than 5%); and four, rare variation position in a population with at least one minor allele with low probability (no more than 5%). Note that the first two of these dynamic positions were combined as MAIR. Overall, the non-redundant dynamic genomics information in VCGDB comprises around 35 million single nucleotide variations (SNVs) that include 29 million rare variations and 0.5 million indels along with annotation information for the genomes. This information covers 1.18% of the positions across the whole human genome (Table 1). The CHB population had more dynamic positions in their genomes than the CHS population, while the CHS genomes had more indels than the CHB genomes. The dynamic information in VCGDB is stored in tables that are organized separately by chromosome. A single table contains a maximum of 7 million dynamic positions, 90 thousand indels, or 5 million rare variations, which ensured the high-efficiency of database searches. Figure 2A shows the distribution of the dynamic positions and indels by their occurrence probabilities in the three pre-defined populations. Major alleles with high probability (>50%) occupy large proportions of the dynamic positions; for instance, major allele with close to 100% probability accounts for 81.3% of the dynamic positions. Figure 2B shows the distribution of indels by their length in the CHN, CHB, and CHS populations. The short indels were more abundant in the genomes than the long indels. Based on the dynamic information in VCGDB, we built the Chinese genome sequences (VCGs) for the CHN, CHS, and CHB populations, which can be used as reference sequences in the currently available alignment software. The three VCGs were validated against the current GRCh37 reference genome using real data from Chinese individuals with higher mapping rates. The results indicated that the VCGs made better templates for Chinese genetic variation analysis than the GRCh37 reference sequence. (See the "Discussion" section for details.)

**Table 1:** Dynamic position counts for the different populations in the virtual Chinese genome database (VCGDB)

	**Number of dynamic positions**		
	**CHN**	**CHS**	**CHB**
**Autosome**	33,780,152	19,591,609	24,109,529
**Heterosome**	1,747,140	937,088	1,222,844
**Chondriosome**	1,006	673	654
**Total**	35,528,298	20,529,370	25,333,027
	**Number of indels**		
	**CHN**	**CHS**	**CHB**
**Autosome**	392,074	454,215	345,647
**Heterosome**	14,360	17,323	12,346
**Chondriosome**	25	20	59
**Total**	406,459	471,558	358,052
	**Number of rare variations**		
	**CHN**	**CHS**	**CHB**
**Autosome**	27,258,690	12,505,097	17,688,051
**Heterosome**	1,477,821	632,253	939,627
**Chondriosome**	907	569	536
**Total**	28,737,418	13,137,919	18,628,214

Position-based annotation information is stored in the entity tables along with basic position information. We carried out a statistical analysis of the dynamic annotation information in different genomic regions, including the exonic regions, intronic regions, 3’UTRs, and 5’UTRs (Figure 2C). We found that 43.97 and 40.59% of the dynamic positions and indels in the VCGDB, respectively, were located in the genetic regions, including the exonic regions, intronic regions, 3’UTRs, and 5’UTRs. To reveal the main differences between the genomes of the pre-defined Chinese populations and the GRCh37 reference genome, we compared the major allele and indel positions against the GRCh37 reference genome to identify the MAIRs. About 6.87% of the dynamics positions and indels were different in the Chinese genomes compared with the GRCh37 reference genome, and this will be considered further in the "Discussion" section. Table 2 shows the results of a statistical analysis of the genetic variations of the MAIR that occurred in the exonic regions. The three pre-defined populations have similar trends of genetic variations. For CHN, synonymous and non-synonymous SNVs were found in 52.80 and 44.24% of the SNVs, respectively, while the stop-gain and stop-loss SNVs totally made up 0.22% of the SNVs. The 1000 Genomes Project Consortium reported 10,000–11,000 non-synonymous variations in an individual genome, which is twice as high as the non-synonymous variations shown in Table 2. The reason may be that the non-synonymous SNVs in Table 2 are intersections of all the non-synonymous SNVs in the genomes of all the individuals in a certain population; therefore, they represent the major variations present in most of the individual genomes, which will definitely be smaller than the variations in each individual genome. The enrichment gene lists for the coding regions that contain the SNVs are presented in Additional file 2: Table S1. Table 3 shows the results of an enrichment analysis of the MAIR that match the locations of GWAS traits that were annotated using the gwasCatalog. The top matches represent the differences between the Chinese populations and the Caucasian GRCh37 reference genome. Clearly, Chinese and Caucasians have distinct physical characteristics; for example, in height (110 hits) and body mass index (34 hits)*.* For the disease-related traits, there were also distinct differences; for example, several common ailments in the Chinese population, such as multiple sclerosis (41 hits), Crohn’s disease (36 hits), coronary heart disease (34 hits) and type 2 diabetes (33 hits), were detected by this analysis. The analysis revealed some differences among the three pre-defined Chinese populations; for example, the CHB population had more hits for the bipolar disorder and rheumatoid arthritis traits than the CHS population, which seems to indicate a small geographical difference between the two populations.

**Table 2:** Statistical analysis of genetic variations in the exonic regions of the Chinese and GRCh37 genomes

	**CHN**	**CHS**	**CHB**
**Synonymous SNV**	6481(52.80%)	6657(52.68%)	6446(52.98%)
**Nonsynonymous SNV**	5430(44.24%)	5609(44.39%)	5362(44.07%)
**Stop-gain SNV**	22(0.18%)	21(0.17%)	22(0.18%)
**Stop-loss SNV**	5(0.04%)	6(0.05%)	5(0.04%)
**Unknown**	336(2.74%)	343(2.71%)	332(2.73%)
**Total**	12,274	12,636	12,167

Genetic variations in the major allele and indel positions against the GRCh37 reference genome (MAIR) were analyzed.

**Table 3:** Enrichment analysis of MAIR in GWAS trait locations in the Chinese and GRCh37 genomes

**Top matches**	**CHN**	**CHS**	**CHB**
**1**	Height (110/324)	Height (108/324)	Height (110/324)
**2**	Multiple sclerosis (41/187)	Multiple sclerosis (41/187)	Multiple sclerosis (41/187)
**3**	Crohn's disease (36/181)	Crohn's disease (40/181)	Crohn's disease (38/181)
**4**	Body mass index (34/109)	Body mass index (34/109)	Coronary heart disease (37/151)
**5**	Coronary heart disease (34/151)	Coronary heart disease (33/151)	Body mass index (36/109)
**6**	Type 2 diabetes (33/164)	Type 2 diabetes (32/164)	Bipolar disorder (32/109)
**7**	Rheumatoid arthritis (31/170)	LDL cholesterol (32/114)	Type 2 diabetes (31/164)
**8**	LDL cholesterol (31/114)	HDL cholesterol (31/118)	Rheumatoid arthritis (30/170)
**9**	Bipolar disorder (30/109)	Type 1 diabetes (29/107)	Bone mineral density (30/87)
**10**	Bone mineral density (30/87)	Bone mineral density (29/87)	LDL cholesterol (30/114)

MAIR, major allele and indel positions, against the reference genome.

**Figure 3:**
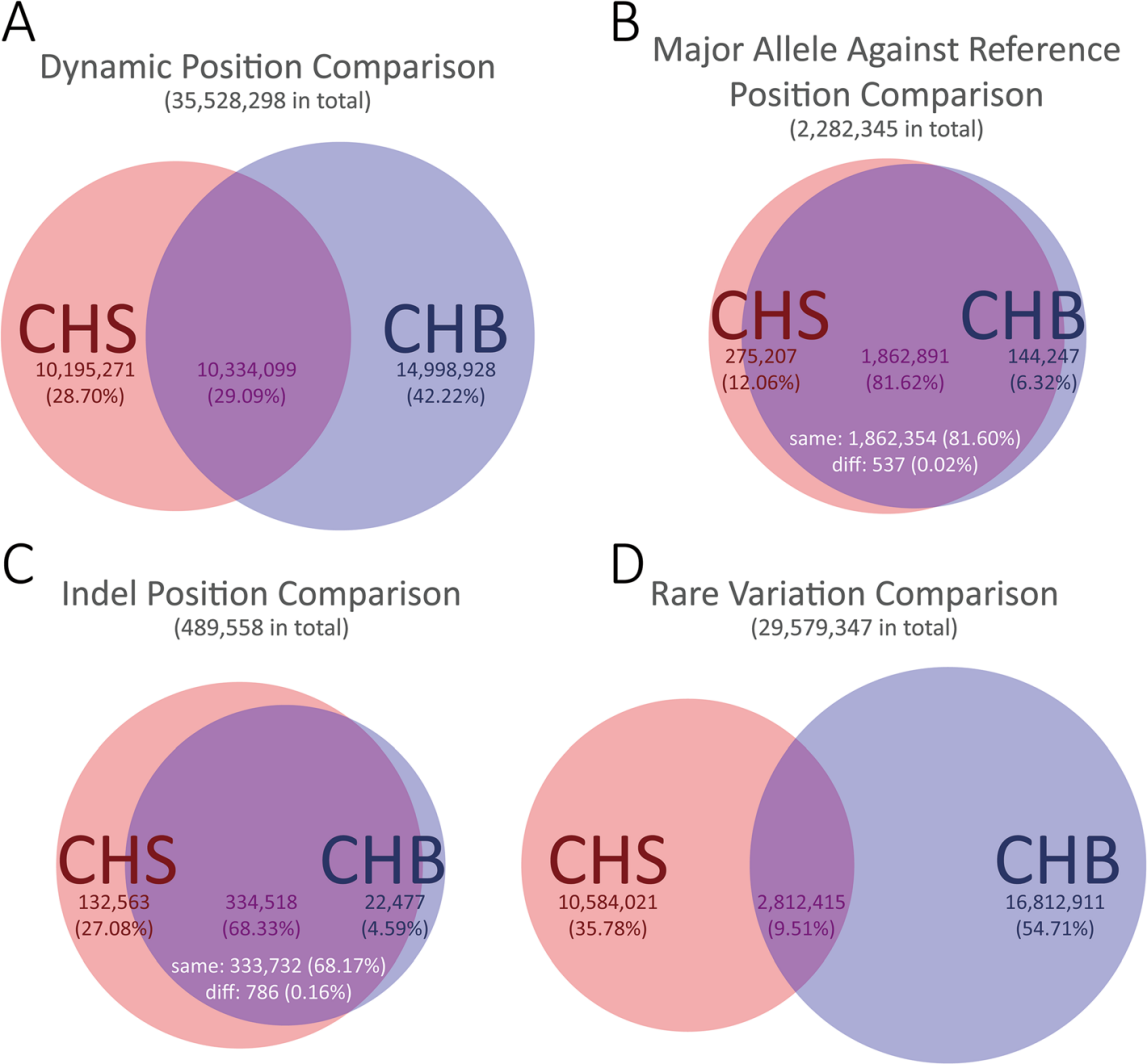
**Differences between the two Han Chinese CHS and CHB populations. A**. Venn diagram of a comparison of the dynamic positions. **B**. Venn diagram of a comparison of major alleles against the GRCh37 reference genome. **C**. Venn diagram of a comparison of high-probability indels against the GRCh37 reference genome. **D**. Venn diagram of a comparison of rare variations. In **B** and **C**, some shared dynamic positions were substituted by the same nucleotides/indels, others were substituted by different nucleotides/indels; these are marked "same" and "diff", respectively.

To find other genetic discrepancy between the CHS and CHB populations, we compared the dynamic information (Figure 3), including the major/minor alleles and indels [41]. We found that there was a low proportion of shared dynamic positions (10,344,099 or 29.09%) in the CHS and CHB populations (Figure 3A), indicating that although these two populations are both Han Chinese, they harbor some genetic mutations. The intersection of dynamic positions between the CHB and CHS genomes is mostly from the MAIR, with a high overlap percentage of 81.60% for the major alleles and 68.17% for the indel positions (Figure 3B and 3C), which is opposite to the trend seen for the dynamic positions (Figure 3A). The dynamic positions come mainly from rare variants (Figure 3D), which also had a low proportion of shared positions (2,812,415 or 9.51%). Compared to the GRCh37 reference genome, the CHS genome had greater numbers of dynamics positions and indels than the CHB genome, which indicates the longer evolutionary distance and relative conservation in the CHS population.

## Utility

VCGDB is a well-organized, highly structured, and indexed database that supports real-time high-performance searches using a web search engine and a genome browser. Users can launch a query to obtain information from several different VCGDB tables, including dynamic position information, MAIR, gene information, and GWAS clinical traits. For example, a dynamic position search will return the basic information on position, population, major and minor allele contribution, and major allele probability and distribution, and so on. A download page is generated so that users can download any Chinese-specific genome sequence generated using the data in the database.

### Web search engine

**Figure 4:**
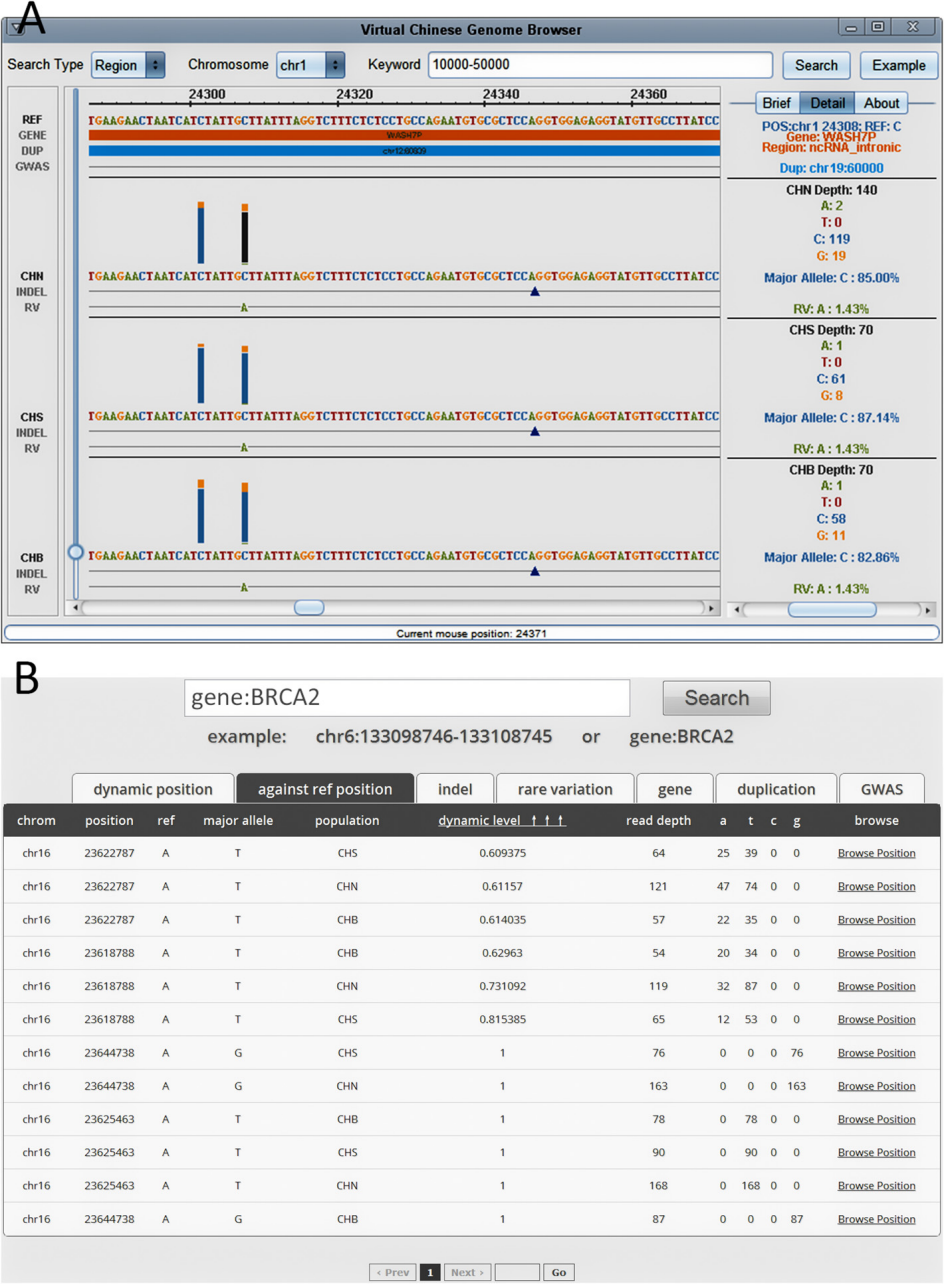
VCGBrowser interface (A) and VCGDB online search page (B).

The VCGDB web search engine (Figure 4B) allows users to input genomic region, gene description, or GWAS trait as keyword searches. The HUGO Gene Nomenclature Committee (HGNC) database was integrated into the search engine to support fuzzy searches that recognize gene symbols or descriptions, rather than simple gene names. It is also a smart search engine that automatically corrects search terms and filters out useless characters. The search results are output in classified tables of detailed dynamic information that support ascending and descending sorting and have symbols like gene name or PubMed ID hyperlinked to the original sources. Moreover, for a more convenience use of our VCGBrowser (described below), a hyperlink is provided at the end of each record that links directly to the browser so that users need not re-enter keywords to visualize genomic regions of interest.

### Virtual Chinese genome browser (VCGBrowser)

The VCGBrowser is a highly interactive user-friendly interface that can be used to view the "virtual" and "dynamic" genomics information (Figure 4A). VCGBrowser is both a web-based applet and a client-based cross-platform application that can be used as an online browser or can be downloaded for use as local software. The VCGBrowser consists of five modules: a control module, browser module, brief module, detail module, and progress module. Unlike traditional genome browsers, VCGBrowser is a creative tool in five main aspects. One, the VCGBrowser allows users to browse the GRCh37 reference genome and the consensus CHN, CHS, and CHB genome sequences in the same window, making it easier for users to detect differences among the populations. Two, the VCGBrowser integrates the dynamic genomics information onto the consensus coordinate of genome sequences. For each dynamic position, multi-colored rectangle bars are used to indicate the nucleotide distribution at that position, triangles are used to indicate indels, and colored characters are used to mark rare variations. Three, the VCGBrowser supports flexible and seamless zooming and browsing. Traditional genome browsers like the UCSC Genome Browser use image-based technologies so that images are always refreshed when zooming in or out [42]. The efficiency of this methodology depends on internet speed and bottlenecks can occur when a large number of users access the site at the same time. VCGBrowser uses servlet technology to fetch all the information and to calculate all the symbols in real-time. Thus, VCGBrowser supports seamless zooming and scrolling to any resolution; from the genomic level that shows the dynamic distribution of a region of interest, to the nucleotide level at which the residues and detailed information can be displayed. Four, the VCGBrowser provides biological annotations, including gene name, gene region, and GWAS traits, and marks them all in the browser window, so that users can easily combine these biological factors with the dynamic genomics information. Five, all the symbols shown in the browser window are selectable and support real-time searching. After a query had been initiated, brief information about the region being queried is displayed in a side window; then, simply clicking on a symbol of interest triggers an instant search in the database, producing detailed information for the users.

### Population-specific reference genomes

A download page is provided for users to download the applications and data in VCGDB. The downloadable version of VCGBrowser can be run on different Windows or Linux operation systems. Moreover, the consensus Chinese reference genome sequences (VCGs) for the CHN, CHS, and CHB populations are also provided on this page. The three VCGs are population-specific linear reference genome sequences that support alignment software, such as BWA, Bowtie, and SOAP [38,39,43].

## Discussion

### VCGDB reflects specific characteristics of the Chinese population

People from different geographical regions have their own specific phenotypes. For instance, Southern Han Chinese (CHS) are generally thinner and shorter, while Han Chinese in Beijing (CHB) are generally stronger and taller. We used the information in VCGDB to examine the potential genotype factors that may lead to these physical differences. Based on the results of a comparison of MAIR in the CHS and CHB genome sequences, we connected the significant genotypic differences that we found with GWAS traits, which showed that these two populations had many specific genotypes related to height and body mass index. These findings might explain why Caucasians generally appear taller and stronger than Chinese. For the disease GWAS traits, Crohn's disease, coronary heart disease, and type 2 diabetes were found to have higher morbidity rates in the Chinese populations, which agreed with a previously reported common ailment investigation [44-46]. Although CHS and CHB are both Han Chinese populations, differences in the dynamic genomics information revealed differences in several disease phenotypes between the two populations.

### VCGDB has higher availability than existing reference sequence in genetic variation analysis of Chinese population

VCGDB is a new type of genome database that revealed possible dynamic variants in the genomes of three Chinese populations. However, it is different from existing reference genomes and its database format is not the traditional linear sequences. Therefore, we used the CHN genome from the VCGs for the genome alignment and genetic variation analysis. To test the performance of the VCGs, we used a real data set to calculate the mapping rate and compared the results against the GRCh37 reference genome and the YanHuang (YH) genome. The real dataset comprised 15 separate samples from Chinese Dai in Xishuangbanna (CDX), Chinese in Denver (CHD), and Japanese in Tokyo (JPT), which are all independent of the data that were used to construct VCGDB. Table 4 shows the results of mapping each of the 15 data samples onto each of Chinese and GRCh37 reference genomes. All the samples mapped to a greater proportion of the VCGs and YH genome compared with the GRCh37 reference genome. Although YH is an individual Chinese male, the 15 Asian genomes mapped to a greater proportion of the VCGs than they did to the YH genome, which confirms the greater ability of the VCGs to represent the genome of the Han Chinese population. This result also reveals the power of large-scale population-based whole-genome sequencing to improve the human reference genome.

**Table 4:** Mapping of 15 Asian genomes onto the VCG, YH and GRCh37 reference genomes

	**CDX samples**	**HG00879**	**HG00844**	**HG00851**	**HG00864**	**HG00879**
**Reference**	GRCh37	46,809,092	54,343,732	58,459,936	41,735,844	46,809,092
		(96.14%)	(95.58%)	(97.78%)	(97.80%)	(96.14%)
	YH	46,963,414	54,578,732	58,711,122	41,929,952	46,963,414
		(96.46%)	(95.99%)	(98.20%)	(98.25%)	(96.46%)
	VCG	47,025,644	54,677,084	58,817,318	42,017,702	47,025,644
		(96.59%)	(96.16%)	(98.37%)	(98.46%)	(96.59%)
	**CHD samples**	**NA18698**	**NA18699**	**NA18126**	**NA18126**	**NA18691**
**Reference**	GRCh37	9,570,212	9,900,966	12,832,078	11,818,302	15,160,036
		(74.52%)	(83.42%)	(70.94%)	(64.55%)	(61.13%)
	YH	9,554,028	9,899,632	12,825,240	11,826,910	15,179,378
		(74.39%)	(83.41%)	(70.90%)	(64.60%)	(61.20%)
	VCG	9,590,720	9,931,332	12,870,814	11,880,666	15,228,994
		(74.68%)	(83.68%)	(71.16%)	(64.89%)	(61.40%)
	**JPT samples**	**NA18988**	**NA18985**	**NA19084**	**NA18984**	**NA18960**
**Reference**	GRCh37	31,213,781	4,235,400	34,476,427	9,353,246	71,376,941
		(89.77%)	(97.06%)	(98.70%)	(96.88%)	(89.93%)
	YH	31,338,365	4,236,640	34,514,636	9,359,975	71,711,466
		(90.13%)	(97.09%)	(98.81%)	(96.95%)	(90.35%)
	VCG	31,410,518	4,236,680	34,500,530	9,361,169	71,898,286
		(90.34%)	(97.09%)	(98.76%)	(96.96%)	(90.59%)

### VCGDB provides a solution strategy for big data

The continuous innovations in high-throughput sequencing technology, even single-molecule sequencing, have dramatically expanded the capacity for description and data collection; however, a large bottleneck remains in the efficiency of compiling, organizing, and manipulating these data. The biggest challenges are in computing resource allocation, parallel computing control, algorithm optimization, and the physical structural design of a database.

VCGDB is considered "virtual" because the reference genome that we provided here does not belong to any real human being; it is the statistical result of terabases of sequencing data from hundreds of Chinese individuals. VCGDB adequately describe the genetic variations, features, and preferences of the Chinese populations that they represent. VCGDB and the associated VCGBrowser provide refined and comprehensive data from the 1000 Genomes Project biological big data, which has been annotated and analyzed with the aim of building connections between human genomic research and medical diagnosis. The VCGBrowser provides a highly flexible user-friendly interface for the user to search and work on. Moreover, users no longer need to deal with massive amounts of data; rather, they can use mature databases and analysis tools to classify individuals or patients into subpopulations that differ in their susceptibility to a particular disease or in their response to a specific treatment. Here, we have developed the VCGDB and VCGBrowser as a support system for researchers and doctors to build an accurate and precise classification of the human genome and diseases, and thereby promote the progress in social healthcare.

The "dynamic" feature in VCGDB can display multiple levels of genetic variation information in the three Chinese populations. Individual genomes were examined first to detect nucleotide-level variations between individuals and populations and to evaluate the degree of variation in the base backbone. Then, all the genetic variation information was collected for all the individuals in the three populations. Finally, the dynamic variations of individual characters and genomic annotation information, such as reference genes, genomic duplications, and GWAS clinical traits were integrated into the VCGDB structure. The data preprocessing that we developed, downsized the raw data to an analyzable scale without losing any detail information, and the analyzing algorithms translated sequencing data into dynamic genomics information using limited time and computing resource. The results are output as a big-table-like data structure, which is convenient for data exporting and follow-up studies. Furthermore, the optimized VCGDB structure allowed the implementation of a high-efficiency real-time search of all the dynamic genomics information in the database along the whole length of the Chinese genomes. The VCGDB structure is a novel database scheme that can be used to deal with the huge amounts of incoming biological data.

### Future developments

Although about 8 terabytes of Chinese genome data have been integrated in VCGDB, it remains just a tiny piece in the huge data iceberg that will be required to fully illustrate the complexities of the human genome. We set a threshold of 5% to define rare variants because of the limited sample size and the current sequencing error rate. Usually, a rare variant that may be disease-related will have an occurrence probability of less than 1% or even one in a million, which is currently almost impossible to detect. In the near future, sequencing projects such as UK10K, The Cancer Genome Atlas (TCGA), and TwinsUK will generate ultra-large volumes of human genome data [47-49]. Because the sequencing data are generated in different laboratories using various platforms, how to normalize these data, merge it with existing data, and analyze and interpret it are big challenges that have to be addressed [19].

Traditional alignment software programs or algorithms always use the static human GRCh37 reference genome as the template and dynamic variations are seldom considered. To overcome this limitation, an advanced dataset could be used in a dynamic mapping process, but this approach wastes data and overlooks potential biological significance. New algorithms with higher mapping speeds and dynamic variation support need to be developed to handle the increasing quantities of data [23]. Conversely, limited computing resources restrain data mining efficiency and its applicability to many investigators. Cloud computing and supercomputing may be the best solution in response to the data explosion crisis [50-52]. We are planning to build a stable data system in the cloud, develop user-friendly tools and pipelines, and establish cloud platforms to accelerate further algorithm development.

Managing and maintenance are critical for databases. We will continue to provide the computational resources, debug the programs, and ensure the stable running of VCGDB and VCGBrowser. In the future, we will continue to monitor the progress of other human genome projects, collect and merge Chinese sequencing samples, execute our data analysis workflow, validate the results, and periodically update VCGDB.

## Conclusions

In this study, we constructed a new type of dynamic genome database of three Chinese populations, including CHS and CHB, which is very different from any of the current traditional human genome databases. VCGDB integrates all the dynamic information generated from the whole-genome sequencing of hundreds of individuals, and combines it with the corresponding genomic annotation information. The "virtual" and "dynamic" features of VCGDB helped reveal genetic variations in the Chinese genomes. We developed a highly interactive user-friendly VCGBrowser, which has significant functions like seamless zooming and real-time searching, for users to search and compare the dynamic information of the different populations in VCGDB. Based on the population-specific information in VCGDB, we build consensus Chinese reference genomes to detect nucleotide preferences in the Chinese populations, and to be compatible with traditional alignment software. We propose that VCGDB offers a feasible strategy for processing big data to keep pace with the growing volume of biological data and provides a robust resource based on the massive amounts of genomics data for genomics studies and investigations into genetic diseases.

### Availability and requirements

**Database homepage:**http://vcg.cbi.ac.cn/

**Requirements:** Java Runtime Environment (JRE) version 1.6.0 or upper

## Abbreviations

VCGDB: Virtual Chinese genome database; VCGBrowser: Virtual Chinese genome browser; SNP: Single nucleotide polymorphism; SNV: Single nucleotide variation; UTR: Untranslated region; MAIR: Major allele and indel positions against reference genome; GWAS: Genome wide association studies; HGNC: HUGO Gene Nomenclature Committee; VCG: Virtual Chinese reference genome; YH: Yan Huang reference genome; CDP: Candidate dynamic position; CHN: Chinese population; CHS: Population from Han Chinese in southeast of China; CHB: Population from Han Chinese in Beijing, China; CHD: Population of Chinese in Denver, Colorado; CDX: Population of Dai Chinese in Xishuangbanna, China; JPT: Population of Japanese in Tokyo, Japan; CEU: Population of Utah residents with Northern and Western European ancestry; YRI: Population of Yoruba in Ibadan, Nigeria.

## Competing interests

The authors declare that they have no competing interests.

## Authors’ contributions

YL and ZJ collected the data. YL, MS and JX designed the workflow. YL, JZ and JW developed the algorithms. YL, MS analyzed the data, constructed the database, and wrote the software. YL and YZ wrote the website codes. YL, JY, JW and JX wrote the manuscript. All authors contributed to and approved the final manuscript.

## Supplementary Material

Additional file 1: Figure S1 Cross-chromosome data extracting logic. **Figure S2.** Database searching and data transferring framework. Click here for file

Additional file 2: Table S1 List of coding region SNVs enrichment gene. Click here for file
