# Supplementary material for: VCGDB: a dynamic genome database of the Chinese population
Source: BMC Genomics. 2014 Apr 5;15:265. doi: 10.1186/1471-2164-15-265 (PMC4028056; doi:10.1186/1471-2164-15-265)
Supplement: Additional file 1: Figure S1 — Cross-chromosome data extracting logic. Figure S2. Database searching and data transferring framework. [file 1471-2164-15-265-S1.DOCX]

**Figure S1. Cross-chromosome data extracting logic**


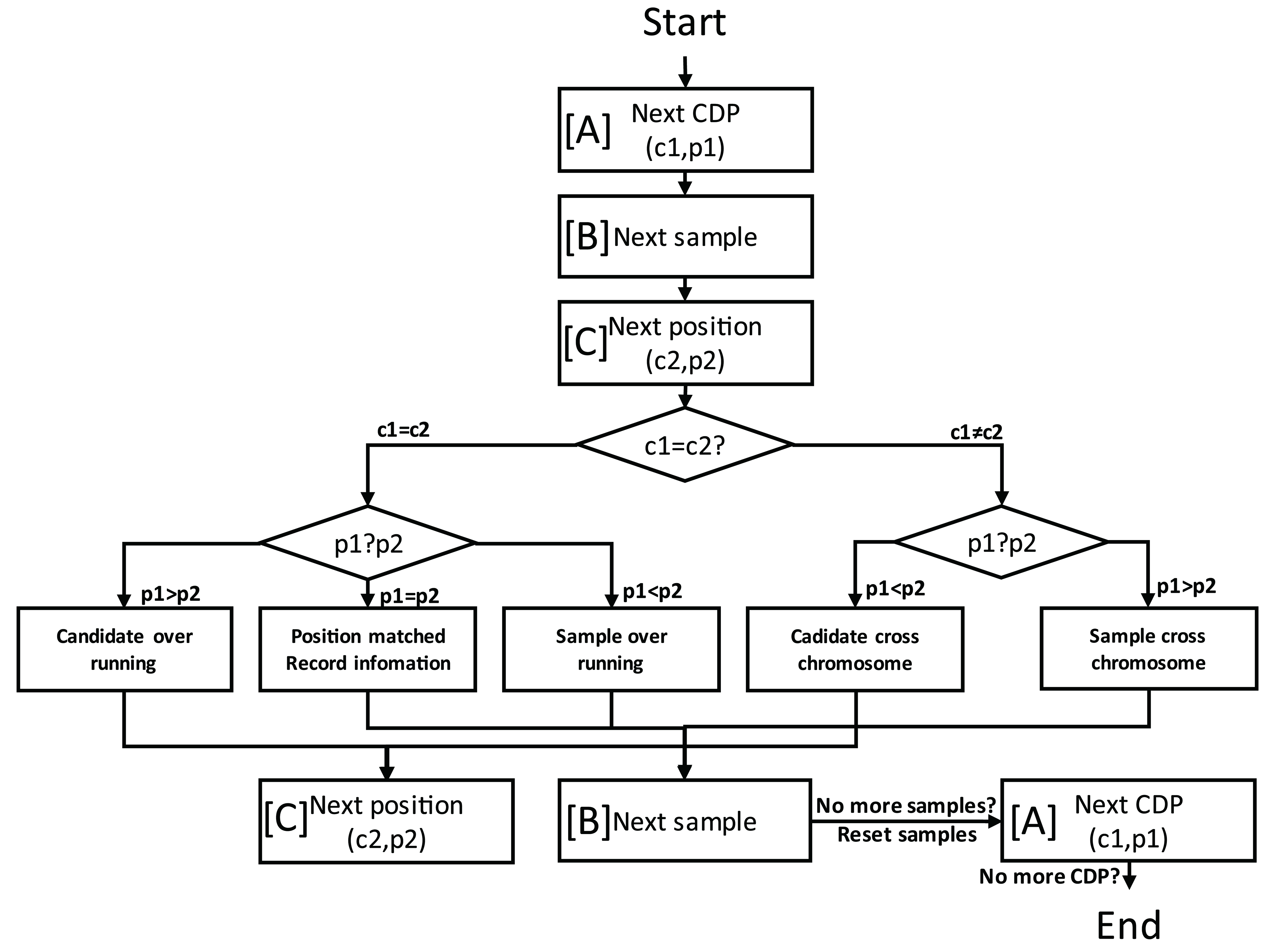


The cross-chromosome data extracting logic is a searching algorithm balancing data extracting time cost and memory use. It inputs the sorted candidate positions and sorted pileup files, use chromosome name and position id to identify and locate positions, control the candidate position reading and sample data looping process, searching, extracting and storing the dynamic information, associated with sample belonging information step by step.

**Figure S2. Database searching and data transferring framework**


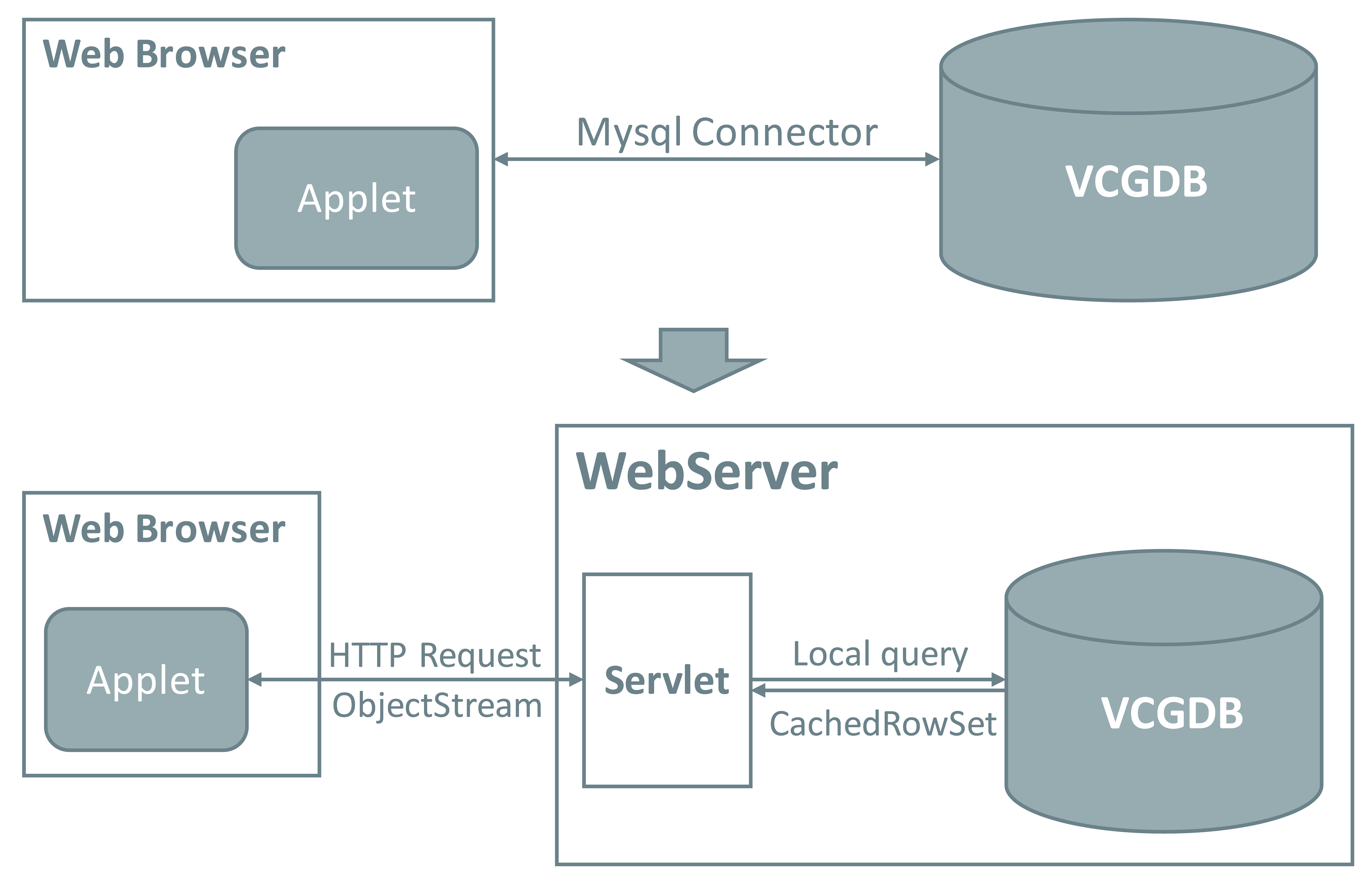


VCGBrowser change the traditional direct database querying structure, and set up an intermediate servlet on the server side. Servlet is set up on the server side where users download the applet, so users do not have to change their local java security policy to run a remote database search, which ensure the data transferring security and simplify the installation of VCGBrowser. Servlet use object based “CachedRowSet” to transfer query result, which ensure the database connection resistance and accelerate network transfer speed.
